# Role of imaging for eligibility and safety of a-NGF clinical trials

**DOI:** 10.1177/1759720X231171768

**Published:** 2023-05-29

**Authors:** Frank W. Roemer, Marc C. Hochberg, John A. Carrino, Andrew J. Kompel, Luis Diaz, Daichi Hayashi, Michel D. Crema, Ali Guermazi

**Affiliations:** Department of Radiology, Universitätsklinikum Erlangen & Friedrich-Alexander-Universität (FAU) Erlangen-Nürnberg, Maximiliansplatz 3, 91054 Erlangen, Germany; Chobanian & Avedisian School of Medicine, Boston University, Boston, MA, USA; University of Maryland School of Medicine, Baltimore, MD, USA; Department of Radiology & Imaging, Hospital for Special Surgery and Weill Cornell Medicine, New York, NY, USA; Chobanian & Avedisian School of Medicine, Boston University, Boston MA, USA; Chobanian & Avedisian School of Medicine, Boston University, Boston MA, USA; Tufts Medical Center, Tufts Medicine, Boston, MA, USA; Chobanian & Avedisian School of Medicine, Boston University, Boston MA, USA; Institute of Sports Imaging, French National Institute of Sports (INSEP), Paris, France; Chobanian & Avedisian School of Medicine, Boston University, Boston MA, USA; Chobanian & Avedisian School of Medicine, Boston University, Boston MA, USA; Boston VA Healthcare System, West Roxbury, MA, USA

**Keywords:** a-NGF, clinical trials, eligibility, MRI, nerve growth factor, osteoarthritis, radiography, safety

## Abstract

Nerve growth factor (a-NGF) inhibitors have been developed for pain treatment including symptomatic osteoarthritis (OA) and have proven analgesic efficacy and improvement in functional outcomes in patients with OA. However, despite initial promising data, a-NGF clinical trials focusing on OA treatment had been suspended in 2010. Reasons were based on concerns regarding accelerated OA progression but were resumed in 2015 including detailed safety mitigation based on imaging. In 2021, an FDA advisory committee voted against approving tanezumab (one of the a-NGF compounds being evaluated) and declared that the risk evaluation and mitigation strategy was not sufficient to mitigate potential safety risks. Future clinical trials evaluating the efficacy of a-NGF or comparable molecules will need to define strict eligibility criteria and will have to include strategies to monitor safety closely. While disease-modifying effects are not the focus of a-NGF treatments, imaging plays an important role to evaluate eligibility of potential participants and to monitor safety during the course of these studies. Aim is to identify subjects with on-going safety findings at the time of inclusion, define those potential participants that are at increased risk for accelerated OA progression and to withdraw subjects from on-going studies in a timely fashion that exhibit imaging-confirmed structural safety events such as rapid progressive OA. OA efficacy- and a-NGF studies apply imaging for different purposes. In OA efficacy trials image acquisition and evaluation aims at maximizing sensitivity in order to capture structural effects between treated and non-treated participants in longitudinal fashion. In contrast, the aim of imaging in a-NGF trials is to enable detection of structural tissue alterations that either increase the risk of a negative outcome (eligibility) or may result in termination of treatment (safety).

## Introduction

Nerve growth factor (NGF) is a neurotrophic protein with numerous pro-nociceptive effects.^1–3^ The role of NGF in perpetuating chronic pain states has been shown by multiple studies in the past, and this has given rise to the development of several NGF inhibitor therapies, although none of these have achieved regulatory approval.^1–3^ The most advanced therapies are NGF-sequestering antibodies, of which several are in later stages of clinical development for the treatment of pain associated with osteoarthritis (OA) or programs that have recently been discontinued.^3–9^

Despite promising efficacy data, anti-nerve growth factor (a-NGF) trials in OA were suspended in 2010 due to concerns over accelerated rates of osteonecrosis in some patients.^10^ However, subsequent interdisciplinary adjudication lead to the conclusion that tanezumab treatment – one of the a-NGF compounds being evaluated at the time – was associated with an increase in rapid progressive osteoarthritis (RPOA).^11^ Specifically, high-dose tanezumab administered in combination with non-steroidal anti-inflammatory drugs (NSAIDs) and pre-existing subchondral insufficiency fractures (SIFs) were risk factors for RPOA in this cohort.^11^ The mechanisms for increased rates of RPOA in the tanezumab program are not fully understood, but several hypotheses have been proposed, including neuropathic and analgesic arthropathy where subjects experiencing efficacious analgesia overuse their joints.^12–16^ The high proportion of adjudicated events of RPOA in participants who were treated with tanezumab in combination with NSAIDs further suggests that other contributing factors may be relevant.^17–19^ In March 2015, the US Food and Drug Administration (FDA) released tanezumab and other a-NGF compounds from clinical hold with the obligation to include stringent mitigation strategies in future phase-III trials including a limitation of a maximum dose of 5 mg. An additional mainstay of these strategies was imaging-based radiographic screening at eligibility and during the course of these studies with the goal to identify subjects with osteonecrosis (ON), SIF, or at risk for RPOA (e.g. those joint with severe malalignment or an atrophic appearance of OA).^18^ The results of this study that included patients with moderate to severe OA of the knee or hip showed statistically significant improvement in pain and physical function outcomes and in patient global assessment of OA, although the overall improvements were modest and tanezumab-treated participants exhibited more joint safety events and total joint replacements.^9^

A biologics license application was submitted in late 2019. In March 2021, an FDA advisory committee voted 19 to 1 against approving tanezumab and declared that the risk evaluation and mitigation strategy were not adequate to address the safety risks. Pfizer Inc. and Eli Lilly and Company announced discontinuation of the tanezumab global clinical development program in October 2021. This decision was based on the regulatory review of tanezumab for the treatment of OA pain by the FDA and the European Medicines Agency.^20,21^

Due to the serious concerns among regulatory authorities regarding the safety profile of NGF inhibitors, other options to block the NGF/ tropomyosin receptor kinase A (TrkA) signaling pathway are being considered.^22^ Some of these non-biological TrkA inhibitors may have advantages over a-NGF compounds, such as in manufacturing, being orally applicable, i.e., more convenient to administer and having shorter half-lives, i.e. easier to adjust the dosage in comparison to the long half-lives of NGF inhibitors.^23^

OA efficacy and a-NGF studies apply imaging for different purposes. While in OA efficacy studies, image acquisition and assessment is optimized for sensitivity to detect structural differences between treated and non-treated participants over time; in a-NGF studies, the aim is to detect structural tissue changes that either increase the risk for an adverse outcome (eligibility) or may result in termination of treatment (safety).^24,25^ In contrast to OA efficacy studies, there is only sparse experience on the role of imaging in a-NGF/TrkA studies.^26^ As a-NGF programs have been worldwide multicenter endeavors with thousands of patients enrolled, multiple expert readers were included to assess the images acquired in mitigation efforts. To achieve high sensitivity to potential adverse events and to homogeneously define eligibility prior to inclusion, potential joint imaging findings need to be carefully defined and illustrated. In addition, extensive reader training and calibration prior to study start-up is paramount. A detailed summary of structural findings of potential safety relevance to the tanezumab and other a-NGF programs has been presented.^27–29^

The aim of this narrative review article is to summarize the experience gained from completed and ongoing clinical trials that employed imaging as part of the eligibility and safety monitoring strategy of a-NGF/TrkA studies. Examples of screening and safety monitoring strategies are primarily based on the tanezumab program but are translatable to other clinical trials with the aim of the modification of the NGF/TrkA signaling pathway.

### Screening and eligibility

The imaging safety strategy in the tanezumab program was based on the development of an illustrative atlas that centralized readers used as the basis for image assessment.^30^ The atlas was applied for subsequent reader training, reader calibration pre- and on-study and for reliability readings on pre-defined image examples. The imaging atlas was developed as an interpretive reading tool for the musculoskeletal (MSK) radiologists (central readers) evaluating subject eligibility and on-study safety in the program.^30^

The imaging atlas’ purpose was to increase consistency among central readers in the following scenarios:

1. Assessment of radiographs applying the Kellgren and Lawrence (KL) scoring system
2. Identifying protocol-defined radiographic risk factors (presence and severity) in potential participants at the time of screening
3. Evaluating radiographs as equivocal and/or defining a reason to order MRI and/or additional radiographs

Parts of the atlas content are publicly available; however, the atlas itself is not.^27–29^

### Exclusionary findings

The primary aim of the radiographic expert evaluation is the identification of shoulder, hip and knee safety findings as defined by study protocols.^27–29^ The site investigator will then have to clinically correlate those imaging findings with the clinical presentation in order to come to an informed decision on subject management.

**Figure 1.**
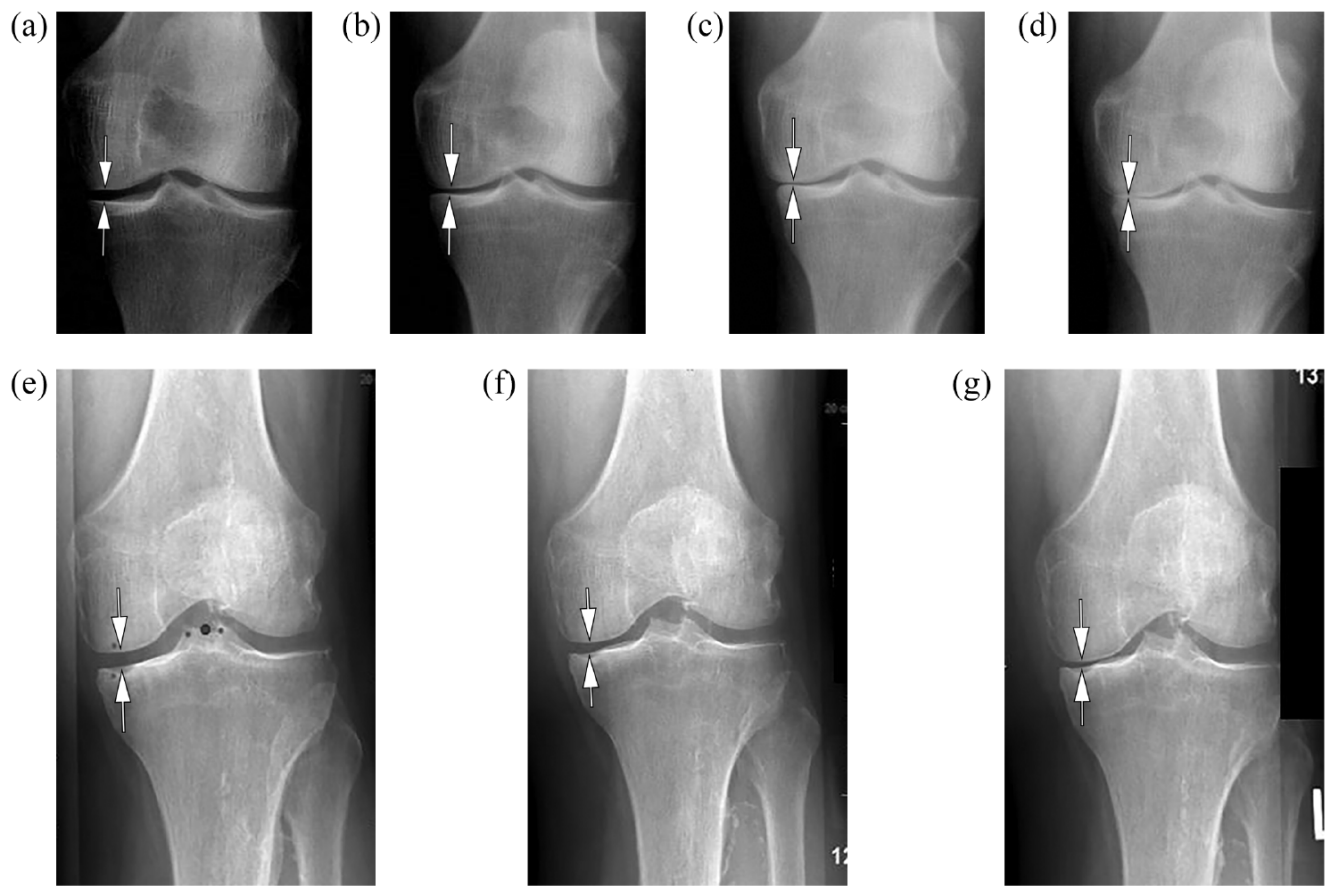
Rapid progressive osteoarthritis Type I (RPOA Type I) over the course of 18 months. (a). Baseline radiograph shows normal medial joint space width (arrows). (b). 6 months follow-up X-ray depicts definite medial joint space narrowing (arrows) with persistent absence of osteophytes medially. (c). Radiograph acquired 8 months after image b shows progressive loss of joint space medially (arrows). (d). 4 months later bone-to-bone appearance with complete obliteration of joint space at the medial compartment is observed (arrows). Figure parts e.-f. show pseudo-progression due to positioning. The image on the left (e) was obtained with 11 degrees beam angulation in order to optimally visualize the medial joint space without bony overlay (arrows). The middle image (f) was obtained using 12 degrees beam angulation resulting in discrete but definitive pseudo-narrowing of the medial joint space (arrows). In screening efforts this knee would be falsely excluded due to appearance of atrophic OA. The right image (g) was acquired with 13 degrees beam angulation, which results in marked medial pseudo-joint space narrowing. These image examples illustrate that minor changes in beam angulation or knee flexion will result in marked changes regarding visualization of the medial joint space. Comparability of X-ray images between serial follow-up visit is paramount in order to correctly assess joint space width changes over time.

There is sparse data available regarding specific risks for program-defined adverse events. Findings such as malalignment of the knee,^31,32^ atrophic OA,^33^ and meniscal injury^34–36^ have been discussed as risk factors for more RPOA, although scientific data-driven reports on RPOA are rare.^37^ Some authors have described radiographic findings consistent with atrophic OA^38^ and, in few circumstances, ON or SIF in cases of RPOA.^39^ No longitudinal data have been published evaluating risk factors that are specific for RPOA, and our knowledge about the disease entity is currently limited. Two different types of RPOA have been defined: RPOA Type 1 is characterized by rapid loss of joint space width (defined as ⩾2 mm in the tanezumab program) within approximately 1 year without evidence of bone loss or destruction.^30^ The diagnosis of RPOA Type 1 can only be established whenever prior images are available that allow for longitudinal assessment. Figure 1 illustrates the role of comparable serial radiographs in order to establish a diagnosis of RPOA Type I and the relevance of correct and repeatable positioning when obtaining the radiographs. The term RPOA Type 2 is used for a condition of abnormal bone loss or articular destruction in a short period, including limited or total collapse of at least one subchondral surface that is commonly not observed in conventional advanced OA.^27^

At screening, readers will assess the glenohumeral, femoroacetabular, and femorotibial joints for radiographic joint safety findings.^30^ Radiographic techniques include Grashey view (frontal oblique) of the glenohumeral joints, anteroposterior (AP) and frog-leg lateral views of the femoroacetabular joints, and the modified Lyon-schuss view (fixed-flexion using SynaFlexer, BioClinica, Newark, CA) of the femorotibial joints.^40^

**Figure 2.**
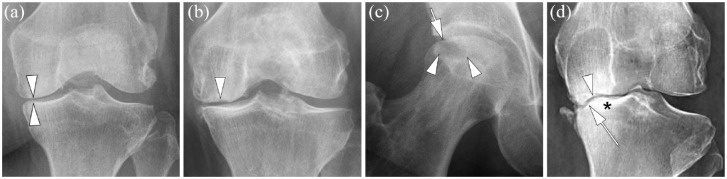
Examples of exclusionary radiographic findings at screening that result in non-eligibility in a-NGF/TrkA programs. (a) Atrophic osteoarthritis (OA). Atrophic OA is defined as a joint with definite joint space narrowing but no or only tiny osteophytes. Anteroposterior radiograph of a left knee shows definite joint space narrowing at the medial tibio-femoral compartment (arrowheads) without osteophyte formation classifying this knee as atrophic osteoarthritis. (b) Subchondral insufficiency fracture (SIF). Radiography is only able to depict the late sequelae of SIF, once definite osteochondral collapse of the joint surface has occurred. Joints with definite SIF at screening will not be eligible for inclusion. Anteroposterior radiograph of a left knee with definite medial joint space narrowing (i.e. Kellgren-Lawrence grade 3) shows medial femoral subchondral cortical depression as the result of SIF (arrowhead). In addition, there is surrounding sclerosis. (c) Osteonecrosis (ON). Early signs of ON are often equivocal on radiographs. Example shows definite ON with an area of subchondral radiolucency (arrowheads) and definite articular collapse (arrow) defining this joint as ON grade 3 according to the ARCO (Association Research Circulation Osseous) classification. (d) Rapid progressive OA Type II. Deformity of the medial femoral (arrowhead) and tibial articular surface (arrow) is noted in this anteroposterior right knee radiograph representing rapidly progressive osteoarthritis Type 2. In addition, there is articular surface collapse and marked subchondral sclerosis (asterisk), especially at the tibial plateau. Consequent varus deformity is observed.

In the tanezumab program, several conditions were defined that possibly result in an increased risk of a joint safety event. Several of these may be radiographically visualized. Unfortunately, it is not possible to prospectively identify all participants who may develop a structural safety finding (e.g. radiographs acquired prior the onset of RPOA are usually normal in appearance): SIF, excessive malalignment of the knee, ON, atrophic or hypotrophic OA, severe chondrocalcinosis, other arthropathies, e.g. rheumatoid arthritis, systemic metabolic bone disease, e.g. primary or metastatic tumors, Paget’s disease, fractures (stress, traumatic, pathologic), and large cystic lesions that may result in an increased risk of fracture. Examples of radiographic exclusionary findings including atrophic OA, RPOA Type 2, SIF and osteonecrosis are presented in Figure 2.

**Figure 3.**
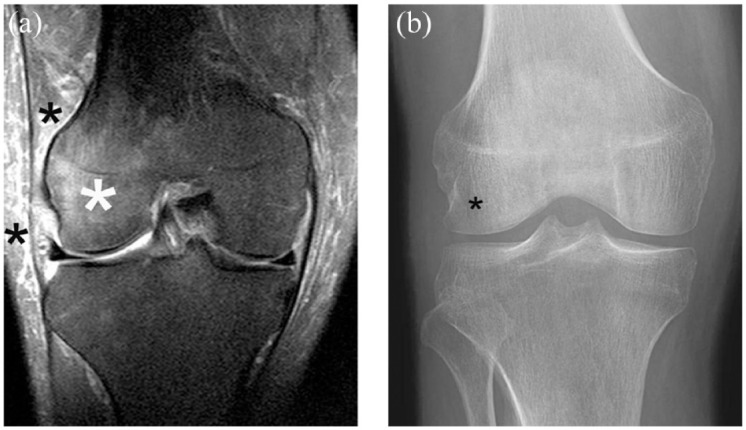
Discordant pain to X-ray. Severe pain in a radiographically normal joint at screening should alert the investigator to further work. Also a sudden increase in pain during the course of the study should result in further exploration regarding the cause of symptom worsening. In this case, primarily so-called for-cause radiographs would be obtained but these may be unremarkable. Additional for-cause MRI is the next step in order to visualize potential x-ray occult structural findings. (a) The image example (coronal fat-suppressed proton density-weighted MRI) show an extensive bone marrow edema alteration in the lateral femoral condyle (large white asterisk). Finding is consistent with idiopathic bone marrow edema syndrome, a disease entity that is strongly associated with severe pain, may be self-limited and heal without sequelae or progress to SIF and structural deterioration. Note additional soft tissue changes between the iliotibial band and in the lateral subcutaneous soft tissues (small black asterisks). These soft tissue alterations are a common accompanying finding. (b) The corresponding radiograph was considered normal at the time of centralized reading. Retrospectively, a subtle diffuse osteopenia in the lateral condyle compared to the medial side may be noted (asterisk). Prior the era of MRI the disease entity was labeled as transient osteoporosis for this reason.

An important reason for exclusion of patients during radiographic screening was disproportionate pain to radiographic findings, defined as a KL grade of 0 or 1 in a knee, hip or shoulder with patient-reported pain of ⩾7 on an 11-point numeric rating scale without other exclusionary radiographic findings.^30^ Disproportionate pain to radiographic findings was not a radiographic exclusion criterion, but patients were deemed ineligible under an exclusion criterion that covered medical conditions that may increase the risk associated with study participation or investigational product administration. Further screening with magnetic resonance imaging (MRI) was not included to determine patient eligibility for the different studies of the program. The rationale for exclusion of patients who had disproportionate pain to radiographic findings was to reduce the risk of enrolling those with radiographically occult conditions such as SIF.^41,42^ An image example of discordant pain to radiographic findings at screening is shown in Figure 3.

### On-study assessment

Once screened successfully, subjects are randomized to a treatment group and followed for the duration of the study. Patients usually undergo radiographic follow-up visits at defined time points, e.g. at 6, 12 and 18 months in a 24-month trial. Readers will not be blinded to chronological sequence and will be assessing serial radiographs at each imaging visit. At each follow-up visit, all prior images will be presented to the readers. Potential joint safety findings will result in a notification of the site investigator.

The presentation of a joint-specific pain score to the reader at the time of image assessment is paramount, as a relevant discrepancy between severe joint pain and discordant radiographic findings will result in the notification of the site investigator (e.g. severe pain in a KL 0 or 1 joint). This may require additional investigation, and an MRI may be requested to further evaluate the possible structural source of pain. Potential incidental findings have been described, but the list may not be inclusive and the individual reader will have to record potential safety findings and communicate those to the study investigator in order to initiate adequate management.

### Role of MRI

**Figure 4.**
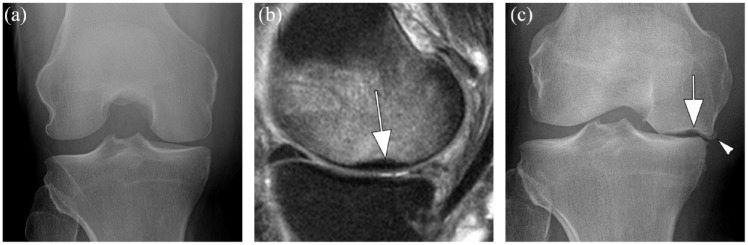
Structurally relevant safety findings may be occult on radiography. (a) anterior-posterior radiograph shows a normal knee joint without surface abnormalities. At the time the patient complained of severe medial knee pain without trauma. (b) Corresponding sagittal proton density-weighted MR image obtained at the same day shows diffuse bone marrow edema in the medial femoral condyle and a focal area of subchondral hypointensity representing subchondral insufficiency fracture (SIF) (arrow). SIF is commonly treated non-surgically including protected weight-bearing. While SIF per se may heal, it may also progress to articular collapse. (c) X-ray obtained 18 months after the initial images were acquired shows an osteochondral defect (arrow), medial joint space narrowing and artciular surface collapse as a result of SIF. In addition there is a small osteophyte that developed at the femoral joint margin (arrohead).

Due to inherent limitations of radiography to detect early disease and morphological changes, for-cause MRIs may be requested in cases of discordance between clinical (incident) pain scores and unremarkable radiographs, which for example, may reveal an underlying diagnosis resulting in a recommendation to discontinue treatment in the event a safety joint endpoint has been reached, especially SIF or ON. An example of radiographically occult SIF is presented in Figure 4.

In the context of eligibility, applying MRI as a primary screening instrument including centralized reading would be ideal to exclude patients with on-going safety findings already prior inclusion or randomization. An alternative approach that has been suggested is MRI acquisition at eligibility but not primarily evaluating those images. Those images would be used for comparison whenever an on-study, so-called ‘for cause’ MRI has been requested and acquired. In this case, the on-study MRI would be compared to the eligibility MRI to confirm whether a given structural finding was truly incident, i.e. developing during the course of the study or potentially had already been ongoing at inclusion but merely not visualized by radiography.

A simplified MRI protocol consisting of sagittal and coronal fluid-sensitive fat-suppressed sequences acquired at 1.5–3 T clinical MR systems will allow ruling out relevant adverse events such as SIF or ON or large areas of bone marrow edema-like signal, which is only detectable on MRI and may be observed in conjunction with OA or cartilage damage but may also represent a stress reaction or idiopathic bone marrow edema syndrome that may increase risk of an adverse event.^43^ In order to apply MRI to large-scale clinical trial endeavors like the a-NGF programs, the feasibility of MRI application is needed. Technological advances in MRI technology have allowed for markedly accelerated image acquisition. These developments include parallel imaging or improvements in 3D acquisitions, which resulted in triplanar MRI of the knee with fluid-sensitive fat-suppressed contrast being acquired in less than 5 min.^44–46^ Artificial intelligence shows additional promise regarding image acceleration.^47,48^

**Figure 5.**
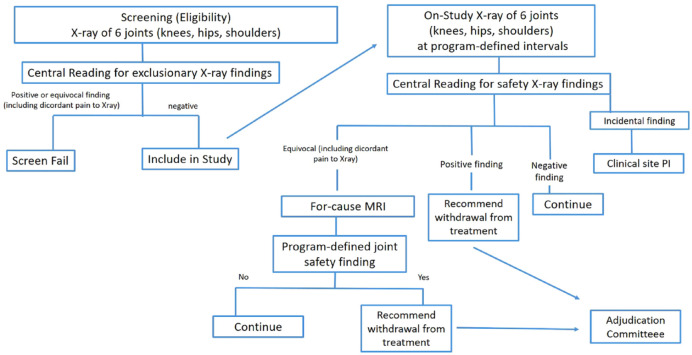
Suggestion for screening and on-study decision algorithm based on potential imaging findings in a-NGF/TrkA programs.

A detailed suggestion for a possible screening and on-study decision algorithm based on potential imaging findings in a-NGF/TrKA programs is presented in Figure 5.

## Discussion

With the release of the clinical hold for the a-NGF programs by the FDA in 2015, several requirements were included to resume clinical trial activities with one of them being close radiologic monitoring for diagnoses at screening and on-study (safety) that may result in increased risk of developing structural adverse events including RPOA Types 1 and 2, SIF and ON. We have described our experiences with an imaging mitigation effort based on the tanezumab program. As experience with a-NGF/TrkA compounds is limited from an imaging perspective, a detailed imaging atlas was created illustrating the more common and but also rare exclusionary findings that may be observed at eligibility or during the course of the different a-NGF/TrkA studies. The basis of the atlas development was the available but limited literature on the topic and the in-depth experience of the MSK radiologists involved in development of the program. Illustrative examples that were included in the atlas have been published previously.^27–29^ Sensitivity concerning eligibility has been reported to range between 0.50 and 0.90 and specificity between 0.40 and 0.83 with NPV between 0.81 and 0.94 and PPV between 0.36 and 0.62.^30^

Experience from previous a-NGF trials has shown that RPOA with either a decline in joint space width of 2 mm or greater within 1 year (RPOA Type 1) or bone destruction and fragmentation beyond what is normally observed in OA (RPOA Type 2), appears to be a safety signal of treatment with monoclonal antibodies to NGF.^49^ Furthermore, the rate of structural joint adverse events was significantly higher in participants treated with tanezumab and concomitant use of NSAIDs and more cases of rapidly progressive OA being observed with higher doses.^10^ It has been discussed, whether the occurrence of RPOA potentially represents a form of neuropathic arthropathy as a result of neural damage including loss of pain perception and impaired joint proprioception.^50,51^ In addition, NSAIDs may have a negative impact on bone healing, which may result in subchondral trabecular fractures that are considered a structural feature present in rapidly progressive OA.^17,52^

In the tanezumab program, the most common radiographically determined reason for patient ineligibility was disproportionate pain to radiographic findings, which was noted for 27% of all radiographically assessed patients and in approximately 10% of knee and hip screening radiographs.^53^ This observation describes a notable absence of OA pathology (relevant particularly for non-target joints), or other pathology (per other exclusion criteria), to explain the extent of pain reported by the patient. Although pain is often misaligned to some extent with radiographic severity of hip and knee OA, in clinical practice such notable discordance would likely warrant further clinical investigation or imaging prior to determining treatment.^54–56^ As the clinical trials of tanezumab solely used radiographic imaging to determine patient eligibility, it was appropriate to exclude these patients from participation. Across the different sets of quarterly central reader testing in the tanezumab program, pairwise reader agreement on overall radiographic eligibility ranged from 72% to 87%, with kappa across all five readers ranging from 0.41 to 0.71. At least four of the five readers agreed on the eligibility status of 73–90% of test cases, and there were no trends for changes in agreement level over time.^53^

A-NGF clinical trials are large studies of up to several thousand participants and several expert readers will usually be involved in centralized image assessment. Only sparse data are available regarding calibration efforts of several radiologic readers in clinical trials and regarding reliability of image assessment in MSK-based clinical trials. At present, no studies are available that have described reliability in regard to exclusionary findings in a-NGF trials. In contrast, reliability of radiographic evaluation for hip and knee OA has been reported previously. One of the largest reading efforts concerning radiographic assessment was centralized scoring of the Osteoarthritis Initiative (OAI) radiographs, where two expert readers assessed images centrally, blinded to each other’s results and all other data. The weighted kappa of agreement between readers was 0.79 for KL grade with adjudication performed by a third reader in cases of disagreement.^57^ In the Dutch CHECK (cohort hip and cohort knee) study reliability between four trained readers was considered substantial for KL assessment and moderate for JSN determination.^58^ The average prevalence-adjusted bias adjusted kappa values (PABAK) regarding KL grading of the knee joint ranged from 0.28 to 0.79, with slightly superior results for assessment of the hip. Increasing the number of readers will likely result in decreased reliability. The imaging atlas was developed as a reference tool for readers, and included image examples help in defining structural OA *versus* no OA or other findings that may be considered somewhat subjective such as moderate or severe chondrocalcinosis, with the latter being an exclusionary finding.

For all a-NGF/TrkA programs, several aspects need to be considered regarding the application of imaging in safety mitigation strategies: Radiography shows most joint findings only relatively late in the course of events and MRI would a much more sensitive instrument to depict early stages of potential adverse events such as SIF or ON.^59–61^ However, the application of MRI for screening or performed routinely while on-study will be a challenge for several reasons. During the course of the study, expert readers will have the possibility to request additional MRI to confirm or rule out suspicious radiographic findings or whenever there are marked discrepancies between no or only minor radiographic findings and the clinical presentation, e.g. severe pain. For this reason, the status of ‘discordant pain to X-ray findings’ has been one of the criteria considered an exclusionary finding at screening in the tanezumab program. Although almost half of the radiographically screened patients were subsequently deemed ineligible, RPOA and protocol-specified joint conditions that could be potential risk factors for RPOA overall were rarely detected on radiographs.

In summary, future a-NGF/TrkA programs will require a sophisticated safety mitigation strategy due to the increased risk of joint safety events that are associated with these findings. One aspect of such risk mitigation is imaging, which is primarily based on obtaining serial radiographs of the large joints at baseline/screening and at defined follow-up intervals during the course of the studies. While X-ray is able to rule out several structural findings, severe clinical symptoms in conjunction with unremarkable X-ray findings may lead to additional acquisition of so-called for-cause MRIs in order to elucidate the origin of symptoms. We have presented an imaging mitigation strategy that may be applicable to any a-NGF/TrkA trial but needs to be tailored to the specifics of each individual program and future regulatory requirements.

## Declarations

**Ethics approval and consent to participate:** Not applicable.

**Consent for publication:** Not applicable.

**Availability of data and materials:** Not applicable.
